# Nonmissile penetrating head injury with a wooden table leg: An illustrative case

**DOI:** 10.1002/ccr3.4057

**Published:** 2021-03-17

**Authors:** Megan M. Finneran, Dario A. Marotta, Emilio M. Nardone

**Affiliations:** ^1^ Neurological Surgery Carle BroMenn Medical Center Normal IL USA; ^2^ Alabama College of Osteopathic Medicine Dothan AL USA; ^3^ Department of Neurology University of Alabama at Birmingham Birmingham AL USA; ^4^ Neurological Surgery Central Illinois Neuro Health Sciences Bloomington IL USA

**Keywords:** intraparenchymal hemorrhage, penetrating head injury, traumatic brain injury

## Abstract

Penetrating head injuries are relatively uncommon and require a unique approach. This report highlights a previously unreported mechanism of injury with a table leg and the steps required to evaluate and promptly treat the patient.

## Graphical abstract

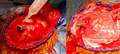

## 1 INTRODUCTION

Nonmissile penetrating head injuries (NMPHI) are rare and vary in terms of severity and outcome. While mechanism of injury can direct management, tailor‐made solutions are often required. We present a 32‐year‐old male patient who sustained a NMPHI from the bolt of a wooden table leg and discuss a focused literature review.

Penetrating head injury (PHI) consists of open head injuries with the introduction of a foreign object into the brain. PHIs are less common than closed head injuries, but typically carry a worse prognosis.^1^ The majority of PHIs consist of ballistic high‐velocity objects in the military setting.^2^ Nonmissile penetrating head injury (NMPHI) is more common in the civilian population and results typically as a consequence of violence, accidents, and suicidal behavior.^3^ NMPHI has been reported involving a variety of objects, including a knife,^1^ welding rod, sewing needle,^4^ nail gun,^2^ glass,^5^ tree branch,^6^ and toothbrush.^7^ As such, severity of injury and treatment modalities range in complexity depending on the mechanism of injury. Herein, we report a unique PHI sustained by a wooden table leg, followed by a review of PHI characteristics contributing to the need for creative treatment approaches.

## 2 ILLUSTRATIVE CASE

### 2.1 History

**FIGURE 1:**
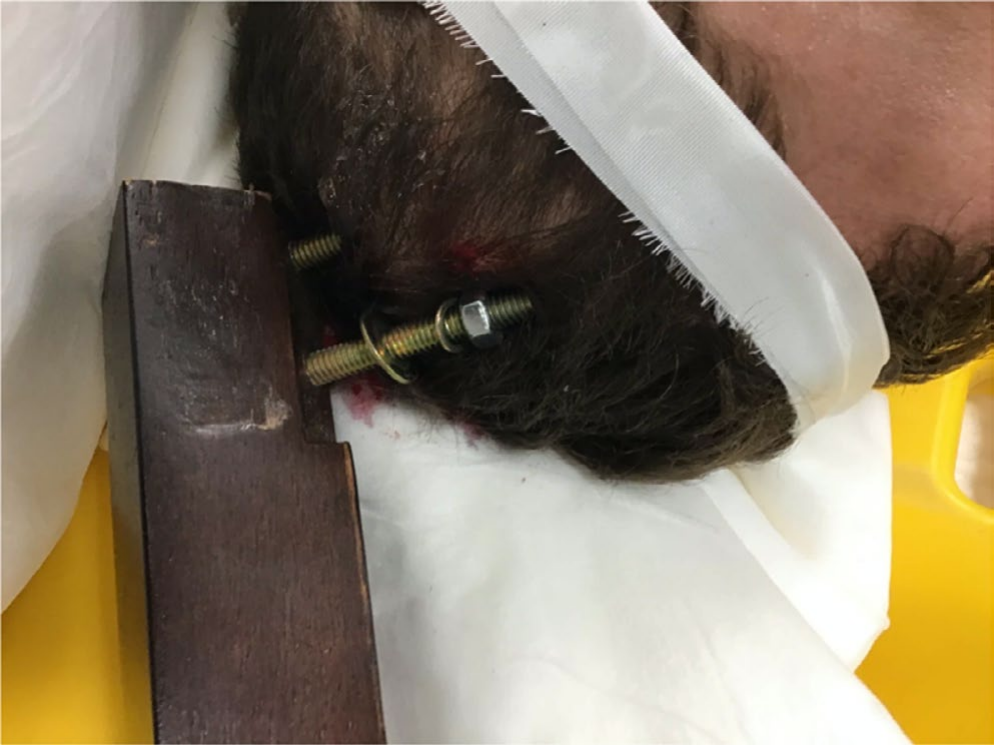
Presentation photograph illustrating metal bolt extending from wooden table leg lodged in the patient's skull

A 32‐year‐old male patient with past medical history of polysubstance abuse and depression presented to a Level 2 trauma center via ambulance after an assault at his residence. Upon arrival, we found a wooden table leg approximately 60 cm in length lodged in his left parietal skull (Figure 1). Details of the assault were unknown.

### 2.2 Examination

On neurosurgical evaluation, the patient was normotensive but tachycardic with a heart rate in the 110s. His airway was intact, and he was spontaneously breathing. His pupils were 2 mm bilaterally and reactive to light. His Glasgow Coma Scale (GCS) score was 13. The patient opened his eyes to voice, followed commands with appropriate motor control in all extremities, but his speech was found to be confused and unintelligible at times. He displayed anomic aphasia, most notably nominal aphasia. Muscle strength testing, according to the Medical Research Council's muscle strength grading system, revealed 4/5 strength in right handgrip with diminished sensation to the right hand.

### 2.3 Diagnostic imaging, testing, and laboratory results

**FIGURE 2:**
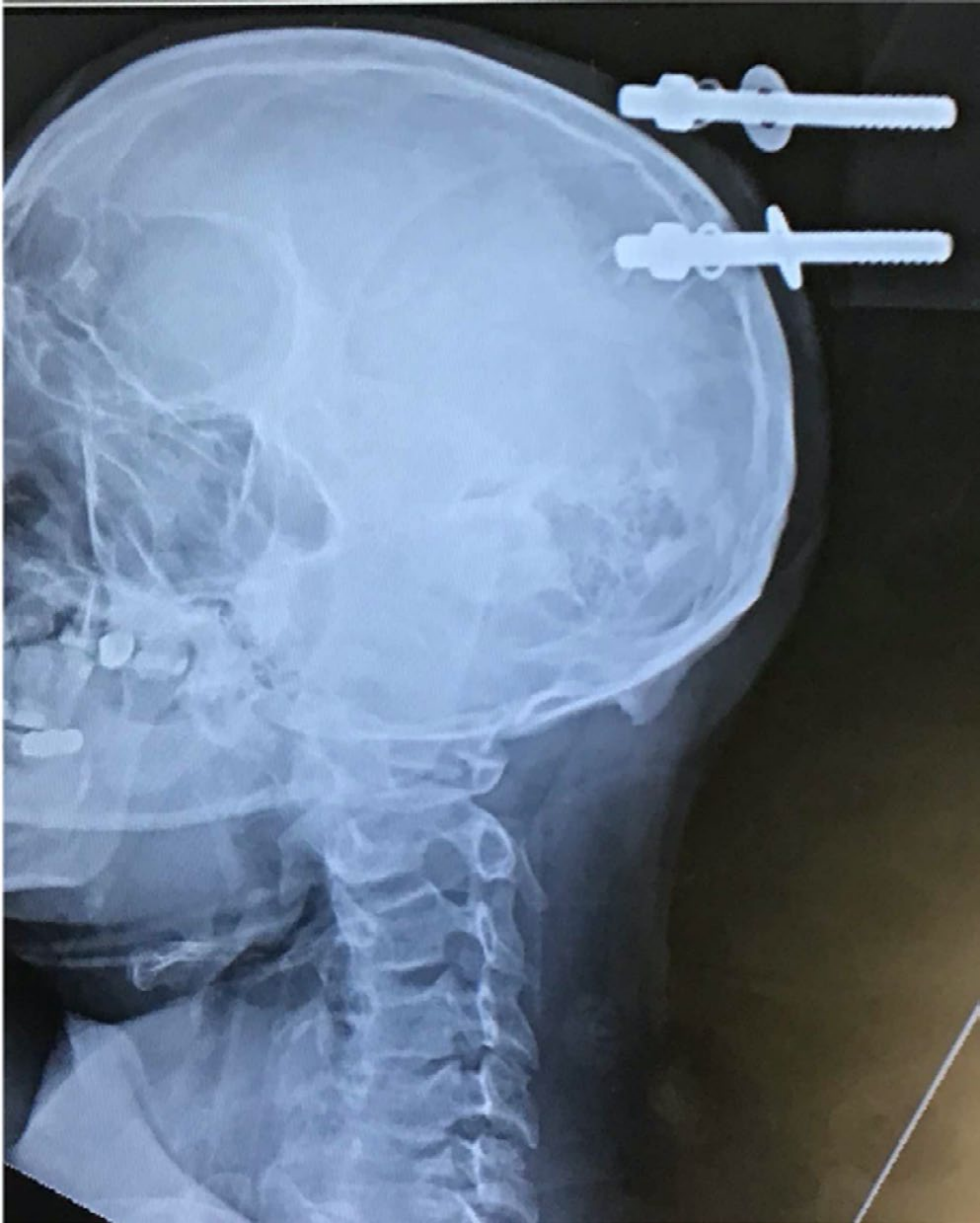
Admission radiograph, portable lateral skull radiograph revealing one bolt embedded in the left parietal skull, penetrating into the brain. The adjacent bolt is superficially abutting the skull

**FIGURE 3:**
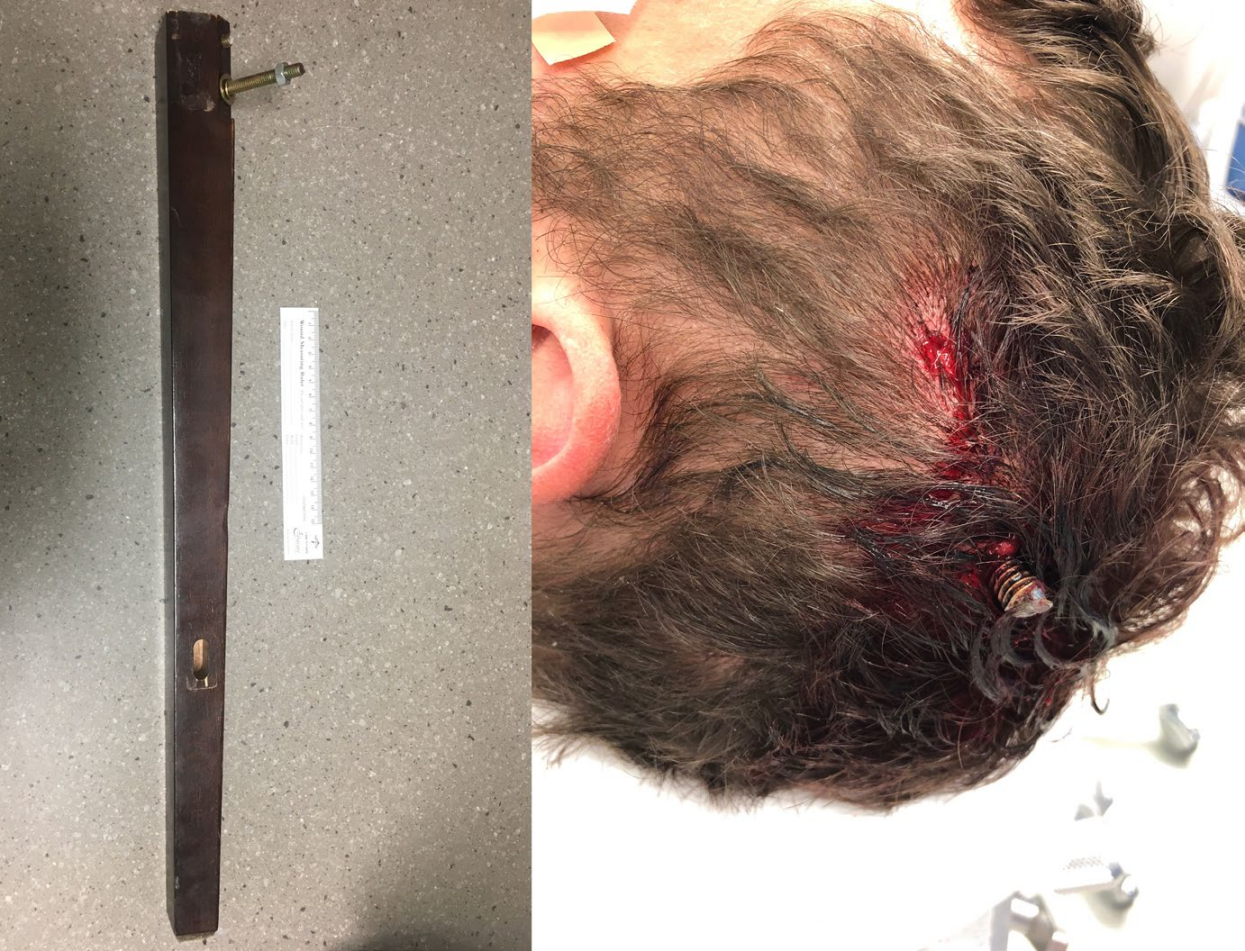
Postmodification photographs, Bolt cutters were used to free the table leg from the patient's skull so he could undergo imaging. Relative size of wooden table leg (left); Remaining bolt penetrating the patient's left parietal bone (right)

**FIGURE 4:**
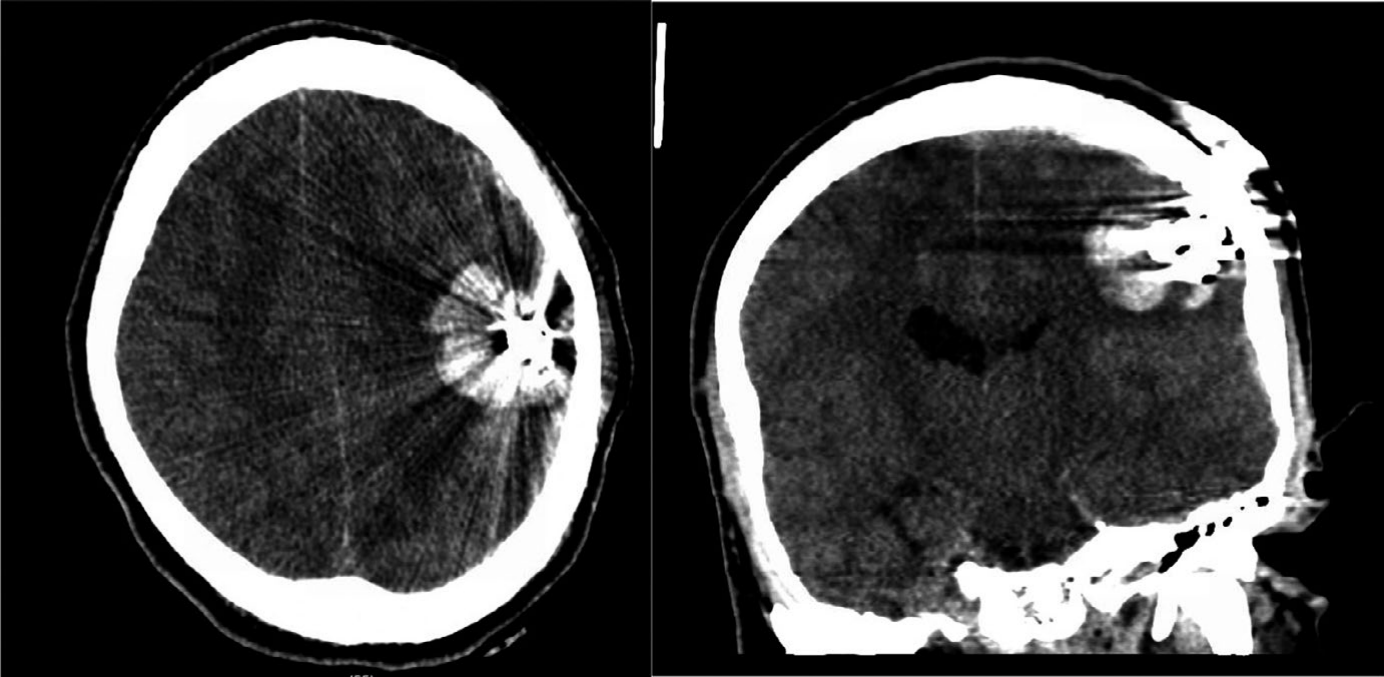
Admission head CT, Axial head computed tomography (left) and coronal head CT (right) demonstrate the bolt penetrating through the left parietal skull into the underlying brain with associated intraparenchymal hemorrhage

A portable skull radiograph revealed a metal bolt, nut, and two washers extending from the wooden table leg and penetrating through the left parietal bone into the underlying parenchyma. A second identical bolt was superficially abutting the scalp (Figure 2). The external position of the table leg relative to the patient's body was such that he was unable to fit within the computed tomography (CT) scanner. A 24‐inch steel bolt cutter was obtained and was used to cut the embedded bolt and detach the table leg (Figure 3). Thereafter, a CT scan without contrast was performed and demonstrated penetration of the bolt into parietal parenchyma amidst multiple small bony fragments within the underlying left parietal lobe. There was also an intraparenchymal hemorrhage measuring 4.0 × 3.7 × 3.0 cm (Figure 4). Computed tomography angiography (CTA) was negative for intracranial vascular injury. CT cervical spine and CT of the chest, abdomen, and pelvis were negative for acute injury. Laboratory findings included a toxicology screen positive for amphetamines, benzodiazepines, methamphetamines, and opiates. Myoglobin was elevated at 183.2 ng/mL (Ref: 25 to 72 ng/mL). Complete blood count and basic metabolic panel were unremarkable.

### 2.4 Treatment and outcome

**FIGURE 5:**
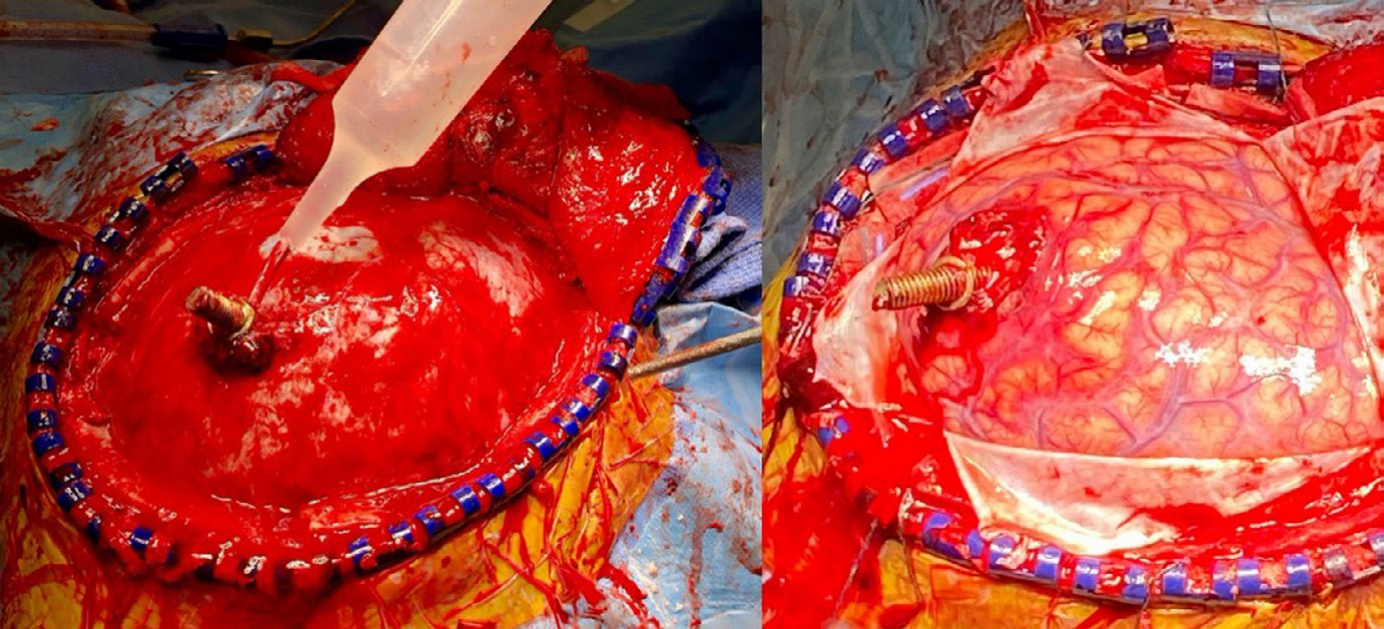
Intraoperative photographs demonstrating scalp retraction and the bone flap elevation while maintaining the bolt in place (left). The dura was opened in a cruciate fashion (right). Copious irrigation was used to extract the bolt and remove the hematoma

The patient was taken to the operating room and positioned supine with a shoulder bump on the side of the injury (left). The head was turned away from the injury (to the right) and secured on a horseshoe headrest. A left‐sided trauma incision was made, with the penetrating object approximately centered in the flap. Four burr holes were placed and connected with a craniotome, with the object remaining in the center of the bone flap. A 12 cm × 15 cm decompressive hemicraniectomy was performed. To minimize further damage, the scalp and bone flap were completely elevated with the bolt in place (Figure 5). The dura was opened in a cruciate fashion around the object (Figure 5). The clot was copiously irrigated such that the bolt was self‐extracted without further penetration. The superficial clot was evacuated. Bovine pericardium was used to loosely close the dura. A subgaleal drain was placed, and the skin was closed.

Postoperatively, the patient received a fitted helmet and was treated with 5 days of intravenous cefazolin 2 g every 8 hours. On postoperative day one, he voiced suicidal ideation in the setting of chronic depression. The patient was evaluated by psychiatry and subsequently started on sertraline 50 mg daily and valproic acid 500 mg twice daily for seizure prophylaxis and agitation. His aphasia gradually improved with subtle persistent word‐finding difficulty. He reported a subjective cognitive delay and demonstrated decreased dexterity in his right hand. On postoperative day two, the subgaleal drain was removed and he was discharged home on postoperative day five with continued occupational and speech therapy for 6 weeks.

Eight weeks postoperatively, the patient was offered cranioplasty with custom‐fitted methyl‐methacrylate cranial prosthesis but ultimately refused.

## 3 DISCUSSION

### 3.1 Observations

Penetrating head injuries can be categorized as high‐ or low‐velocity injuries. The definitive difference between the two is debated and generally considered to be characterized by the nature of the injury rather than the numerical velocity.^8^ High‐velocity injuries create damage beyond the immediate point of contact, whereas low‐velocity injuries cause localized damage along the trajectory of the penetrating object.^9^ As such, this was a case of low‐velocity injury sustained by a blunt metal bolt affixed to a wooden table leg wielded by an assailant. The amount of force required to penetrate the skull with a low‐velocity injury varies by location. The thickest parts of the skull are the posterior parietal and occipital bones, followed by the temporal and frontal bones.^10^ The force needed to penetrate the scalp varies by many factors but is estimated to be approximately 49 N, while 540 N is needed to penetrate the parietal bone.^11^

Due to the diverse nature of PHIs, treatment algorithms are not well‐defined. Treatment primarily consists of resuscitation as needed, including controlling persistent bleeding and intracranial hypertension with life‐saving measures. Antitetanic vaccine should be administered.^1^ A thorough examination should be performed, including of the skin and penetration site as well as a neurological examination.

Blind removal of an object is not recommended as it may cause further damage to neural tissue and adjacent vessels.^12^ This would have been particularly problematic in this case given the two washers extending circumferentially from the threads of the bolt within the brain parenchyma. Penetrating injury to the skull carries a worse prognosis when the object is extracted by the assailant due to the seesaw movement during removal and the release of the tamponade effect of the object on an potentially injured vessel.^13^

Imaging is essential to formulate a proper approach to PHIs. Plain radiographs and noncontrast head CTs are the most appropriate initial tests.^14^ In this case, the angle and length of the embedded table leg prohibited imaging other than a portable radiograph since the patient could not fit into a CT scanner. We located a bolt cutter and, taking caution to minimize movement of the object, cut the distal portion of the embedded bolt. Importantly, magnetic resonance imaging (MRI) is not recommended in cases of PHI due to unknown composition of foreign bodies and the potential for retained metal. In examining the radiograph, we were able to identify that one bolt superficially abutted, but did not invade, the skull. We recommend obtaining a CT prior to entering the operating room. In this case, it demonstrated a large underlying hemorrhage. As a result, we performed a large decompressive craniectomy rather than a small procedure to simply remove the foreign body. CTA should also be performed to rule out vascular injury. Digital subtraction angiography (DSA) can be considered as well, particularly when metal foreign bodies create significant artifact that obscure CTA.^12^

There is a paucity of literature regarding the decision for craniotomy compared to craniectomy for PHI.^15^ In this case, craniectomy was selected for a multitude of reasons. The bone flap was contaminated by the foreign bolt, prompting us to discard it. Additionally, the patient's relatively young age and healthy brain compounded with the underlying hemorrhage raised concern for the development of edema. For this reason, a large craniectomy was performed. The decision for placement of burr holes and shape of the craniotomy also vary according to the size and shape of the foreign object. Some authors suggest a D‐shaped craniotomy, with burr holes placed on either side of the object and forming the flat segment of the D.^16^ In this case, we elected to perform a traditional trauma craniectomy centered around the shaft of the bolt. Its round shape allowed for easy removal of the bone with minimal motion of the penetrating object itself.

Complications arising from PHI are as variable as the foreign objects which cause them. Multidisciplinary management of these injuries, specifically including neurosurgery, otolaryngology, and neuroendovascular surgery, has shown positive outcomes.^2^ The most common secondary injuries include damage to nearby vascular structures and infection.^17^ Thus, CT imaging should be read expeditiously with prompt initiation of bacterial prophylaxis with gram‐positive coverage, such as cefazolin. Patients can also experience posttraumatic seizures and epilepsy^18^ which may warrant seizure prophylaxis, such as valproic acid. Long‐standing changes in personality, cognition, and speech are commensurate with severity and location of the originating insult.^19^, ^20^ While increasing severity of traumatic brain injury corresponds to greater cognitive and functional impairment, immediate postinjury rehabilitation is recommended to reduce short‐ and long‐term consequences of these potentially devastating head injuries.^21^

### 3.2 Lessons

This case of penetrating head injury in a healthy 32‐year‐old with the bolt of a wooden table leg to the left parietal skull and underlying brain parenchyma is the first such case reported in the literature. PHIs vary vastly with regards to mechanism, foreign object, and extent of injury. The neurosurgeon must be prepared to formulate a creative approach to each unique case to safely remove the object and minimize the risk of complications.

## AUTHOR CONTRIBUTIONS

MMF: Composed original manuscript, gathered data from the case including images. DAM: Edited article, enhanced manuscript. EMN: Oversaw case, edited final manuscript.

## CONFLICT OF INTEREST

The authors report no conflict of interest.

## Data Availability

Data sharing is not applicable to this article as no datasets were generated or analyzed during the current study.
